# Optimizing PGRs for in vitro shoot proliferation of pomegranate with bayesian-tuned ensemble stacking regression and NSGA-II: a comparative evaluation of machine learning models

**DOI:** 10.1186/s13007-024-01211-5

**Published:** 2024-05-31

**Authors:** Saeedeh Zarbakhsh, Ali Reza Shahsavar, Mohammad Soltani

**Affiliations:** 1 Department of Horticultural Science, College of Agriculture, Faculty of Agriculture, Shiraz University, Shiraz, Iran; 2 Independent Researcher, Tehran, Iran

**Keywords:** Hyperparameter tuning, Machine learning, Evolutionary optimization algorithm, Plant growth regulators, Pomegranate in vitro propagation

## Abstract

**Background:**

The process of optimizing in vitro shoot proliferation is a complicated task, as it is influenced by interactions of many factors as well as genotype. This study investigated the role of various concentrations of plant growth regulators (zeatin and gibberellic acid) in the successful in vitro shoot proliferation of three *Punica granatum* cultivars (‘Faroogh’, ‘Atabaki’ and ‘Shirineshahvar’). Also, the utility of five Machine Learning (ML) algorithms—Support Vector Regression (SVR), Random Forest (RF), Extreme Gradient Boosting (XGB), Ensemble Stacking Regression (ESR) and Elastic Net Multivariate Linear Regression (ENMLR)—as modeling tools were evaluated on in vitro multiplication of pomegranate. A new automatic hyperparameter optimization method named Adaptive Tree Pazen Estimator (ATPE) was developed to tune the hyperparameters. The performance of the models was evaluated and compared using statistical indicators (MAE, RMSE, RRMSE, MAPE, R and R^2^), while a specific Global Performance Indicator (GPI) was introduced to rank the models based on a single parameter. Moreover, Non‑dominated Sorting Genetic Algorithm‑II (NSGA‑II) was employed to optimize the selected prediction model.

**Results:**

The results demonstrated that the ESR algorithm exhibited higher predictive accuracy in comparison to other ML algorithms. The ESR model was subsequently introduced for optimization by NSGA‑II. ESR-NSGA‑II revealed that the highest proliferation rate (3.47, 3.84, and 3.22), shoot length (2.74, 3.32, and 1.86 cm), leave number (18.18, 19.76, and 18.77), and explant survival (84.21%, 85.49%, and 56.39%) could be achieved with a medium containing 0.750, 0.654, and 0.705 mg/L zeatin, and 0.50, 0.329, and 0.347 mg/L gibberellic acid in the ‘Atabaki’, ‘Faroogh’, and ‘Shirineshahvar’ cultivars, respectively.

**Conclusions:**

This study demonstrates that the 'Shirineshahvar' cultivar exhibited lower shoot proliferation success compared to the other cultivars. The results indicated the good performance of ESR-NSGA-II in modeling and optimizing in vitro propagation. ESR-NSGA-II can be applied as an up-to-date and reliable computational tool for future studies in plant in vitro culture.

## Background

**Fig. 1:**
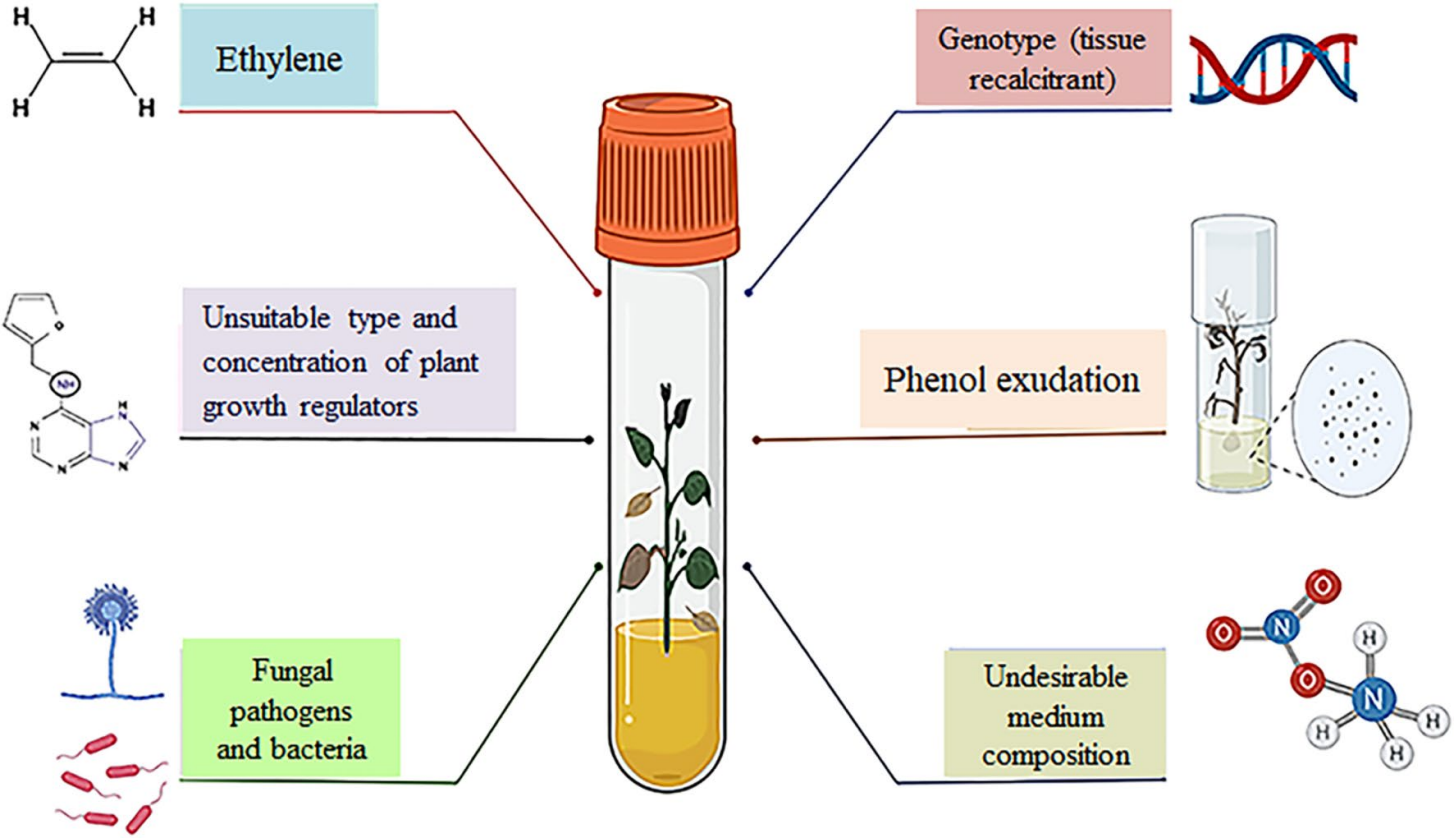
A schematic view of different factors that influence physiological disorders of in vitro plants

Over the past decade, the pomegranate tree (*Punica granatum* L.) has attained significant attention as an economically super fruit cultivated throughout the world, particularly in the arid and semiarid regions. This is due to its high medicinal effects, rich content of bioactive compounds such as antioxidant polyphenol, and numerous health advantages [1, 2]. Traditional methods of propagating pomegranates include sexual propagation through seeds and vegetative methods. However, both conventional propagation methods may face several limitations that cause pomegranate propagation to be difficult. Vegetative methods are time-consuming, dependent on seasonal production, and require intensive labor. Moreover, a large number of plants derived from cuttings often fail to survive [3]. On the other hand, sexual methods are challenging due to the high heterozygosis and a long juvenile period in plants. In addition, seedlings propagated by mentioned methods are strongly affected by pest infestation and diseases [4]. So, to achieve large-scale pomegranate cultivation, in vitro cell and organ culture techniques have been developed. Plant tissue culture methods offer a promising approach for the rapid production of true-to-type pomegranate plants and the biotechnological exploitation of pomegranate and other plant species with valuable properties [5]. Previous studies have attempted to apply in vitro culture techniques to propagate different cultivars of pomegranate [6, 7]. However, the findings have clearly emphasized that pomegranate micropropagation is moderately difficult and can vary depending on the cultivar, probably due to genetic variations among them [6, 8]. Nevertheless, the successful propagation of economically important woody plant species like pomegranate still presents challenges, due to the emergence of some problems during the proliferation stage including defoliation of explants, shoot tip necrosis, callusing, and hyperhydricity. These plant physiological disorders arise from factors such as undesirable medium composition, unsuitable type and concentration of plant growth regulators (PGRs), microbial contamination, phenolic browning caused by phenol secretion, ethylene accumulation, and tissue recalcitrance to proliferation (Fig. 1) [8–10].

The successful in vitro propagation of fruit trees is an intricate process that is influenced by numerous factors, including culture conditions, plant materials, and the composition of culture media, particularly PGRs [11]. Extensive research has emphasized the crucial role of PGRs, such as cytokinins and auxins, and their different combinations with gibberellic acid (GA_3_) in promoting shoot regeneration in different pomegranate cultivars [7]. However, certain PGRs have shown varying levels of effectiveness in promoting proliferation. For example, 6-γ,γ-dimethylallylaminopurina (2-iP) has been reported to have lower proliferative efficiency, while others like 6-Benzylaminopurine (BAP), a commonly used cytokinin in tissue culture, can produce short and thin shoots, sometimes accompanied by excessive callus proliferation. Among the cytokinins, zeatin (ZT), a natural cytokinin, has been found to play a vital role in stimulating the maximum axillary buds and is applied at various concentrations either alone or in combination with other growth regulators. ZT is considered desirable for its stability in nutrient media, as it does not easily degrade or break down, thus providing sustained benefits for rapid and high rates of proliferation in most plant explants [12, 13]. Although different growth regulators, including BAP, kinetin, thidiazuron (TDZ), GA_3_, and IBA, have been used in various combinations with or without ZT to promote the stimulation of axillary buds, GA_3_ is particularly known for inducing rapid shoot elongation, which is beneficial for subsequent rooting. Considering the high cost of ZT, researchers are actively exploring the combined use of ZT with other cytokinins while maintaining the proliferative potential of shoot cultures [14]. However, it is important not to overlook the role of ZT in ensuring a good rate of proliferation [12]. Nonetheless, it is crucial to acknowledge that the responses of different pomegranate cultivars to in vitro propagation are significantly vary depending on the interacting factors during the in vitro process, even in closely related species [15]. Therefore, to achieve optimal results, optimizing of specific in vitro culture condition is necessary for each cultivar.

In vitro micropropagation is a multifactorial and complex biological process influenced by genotype/cultivar and various interacting factors that are crucial for optimizing this process. Traditional statistical techniques encounter with significant challenges in deciphering the large datasets of biological interactions, especially when datasets are nonlinear, complex, noisy, and ambiguous in nature, as observed in in vitro culture processes [16]. To overcome these challenges, advanced computer-based technologies such as Machine Learning (ML) tools have emerged as capable solutions for analyzing and predicting complex and multivariate datasets with high accuracy. ML approaches offer the advantage of autonomous learning and data transformation into useful information without being humanly programmed [17]. Recent studies have highlighted the superior predictive performance of MLs over traditional statistics in various in vitro culture systems, including optimizing culture conditions for shoot proliferation and rooting [10, 18, 19], androgenesis [20], seed germination [21], somatic embryogenesis [22], gene transformation [23], and enhancing of the secondary metabolite biosynthesis [24].

Among the various algorithm-based ML tools, ensemble learning methods have gained significant attention due to their simplicity and their ability to create powerful and robust predictions. These methods can be broadly categorized into bagging, boosting, and stacking/blending. Notably, three prominent ensemble learning methods are Extreme Gradient Boosting (XGB), which utilizes the boosting concept, Random Forest (RF), based on bagging concept, and Ensemble Stacking Regression (ESR), based on stacking concept [25]. Support Vector Machine (SVM) is a robust ML method that has been widely recognized for its remarkable accuracy in plant in vitro micropropagation, as evidenced by the findings of previous studies [19, 26]. One notable advantage of SVM is its ability to effectively handle high-dimensional data without encountering difficulties. Researchers have explored the potential of SVM to address the challenges by utilizing a small training dataset, further highlighting the versatility and effectiveness of SVM in providing accurate and reliable predictions even with limited training data [27]. The Elastic Net Multivariate Linear Regression (ENMLR) was introduced by Zou and Hastie [28] as a robust approach for analyzing high-dimensional datasets. It was designed to overcome the limitations of the LASSO method. By incorporating regression techniques, ENMLR effectively regularizes and selects important predictor variables, thereby improving prediction accuracy of sparse modeling. This method has demonstrated its value in addressing the challenges associated with multicollinearity among predictor variables [29]. Selecting the most appropriate ML method depends on the association between input and output variables, as well as the optimization of hyperparameters [19]. In addition, the combination of ML techniques with evolutionary optimization algorithms confers significant advantages in predicting the critical factors that influence plant growth parameters in in vitro culture systems. One powerful algorithm in this regard is the non-dominated sorting genetic algorithm-II (NSGA-II), which is widely recognized as a search algorithm for optimizing multi-objective problems. NSGA-II enables efficient solving and prediction of complex processes while providing a simplified interpretation of results, simultaneously [30]. In previous studies, the combining approach of ML with NSGA-II (ML-NSGA-II) has been acknowledged as a robust modeling technique for complex datasets, such as in optimizing the protocol of in vitro tissue culture on micropropagation phases [21, 31, 32] and in various plant science fields [30, 33].

Based on our current knowledge, the application of ML algorithms as a novel strategy for modeling and predicting the in vitro shoot proliferation of pomegranate plants remains largely unexplored. The overall objective of this study is (i) to evaluate the effects of ZT at different concentrations and in combination with GA_3_ on optimizing the tissue culture protocol of three commercially significant cultivars, namely ‘Faroogh’, ‘Atabaki’ and ‘Shirineshahvar’; (ii) to compare the potential robustness of the most commonly used ML algorithms, including SVR, RF, XGB, ESR, and ENMLR, in terms of their ability to model and optimize of the in vitro shoot proliferation process of pomegranate cultivars; and (iii) to employ the NSGA-II in order to predict the most effective level of PGRs for enhancing the proliferation of pomegranate. To our knowledge, this study is the first application of ML models for optimizing pomegranate tissue culture media. In addition, despite the potential advantages of ESR and ENMLR, no study has been conducted on applying these procedures in plant science.

## Materials and methods

### Plant material and explant preparation

**Fig. 2:**
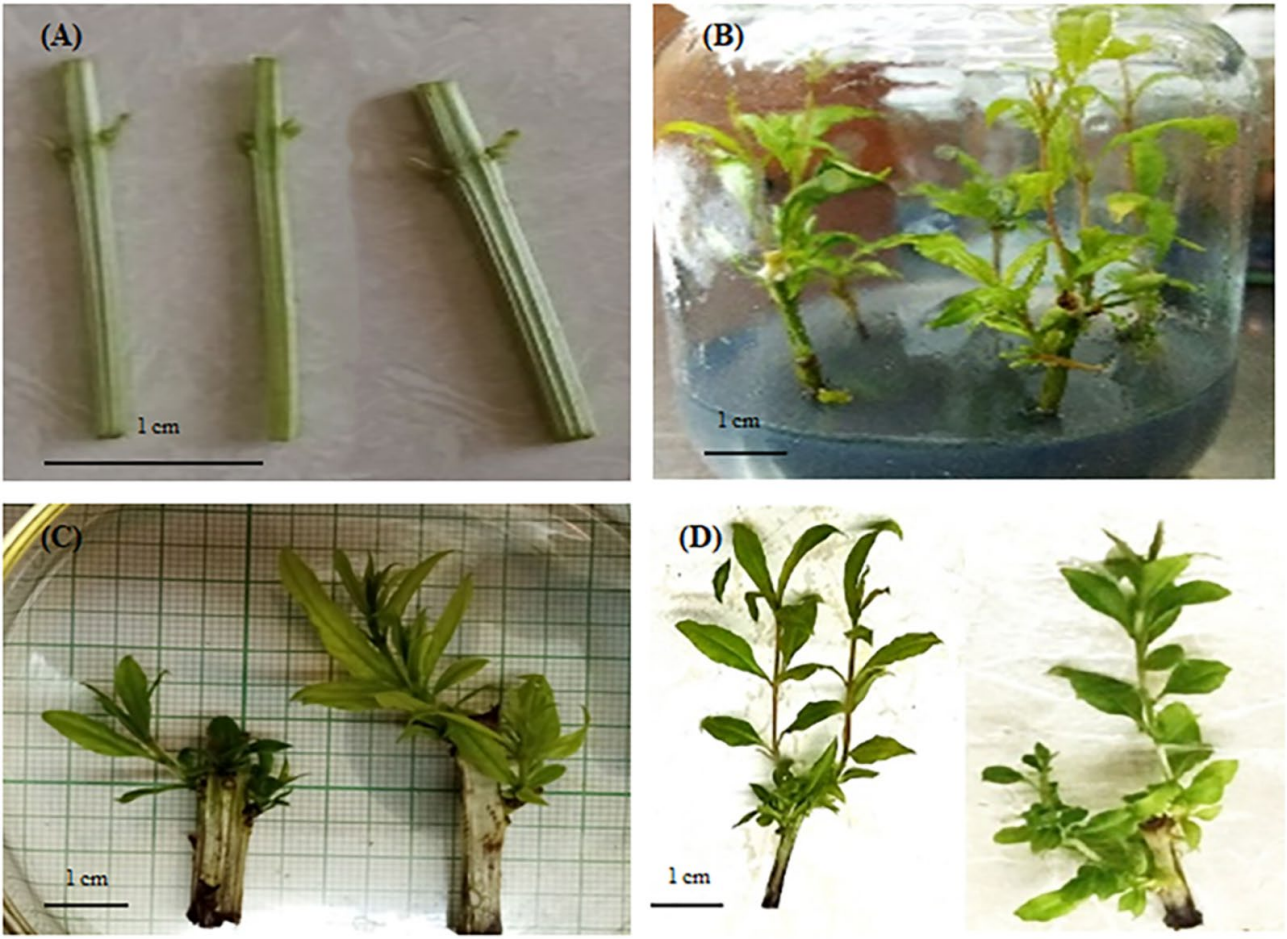
In vitro propagation of pomegranate cultivar ‘Faroogh’. **a** Single-node explants, **b** shoot proliferation in mMS medium supplemented with 0.750 mg/L zeatin and 0.500 mg/L gibberellic acid, **c** shoot proliferation in control medium, and (**d**) shoots propagated in mMS medium supplemented with 0.750 mg/L zeatin and 0.500 mg/L gibberellic acid

The experiments were conducted using single nodal explants from three different pomegranate cultivars: ‘Faroogh’, ‘Atabaki’ and ‘Shirineshahvar’. These explants were obtained from pomegranate plants grown in a greenhouse of College of Agriculture, Shiraz University, Iran. Explants were pre-sterilized using a liquid soap solution and rinsed several times with tap water. Subsequently, the explants were subjected to surface sterilization by immersing them in 70% aqueous ethanol for 30 s, followed by treatment with 5% sodium hypochlorite for 10 min. Afterward, the explants were washed three times with sterilized distilled water under a laminar airflow chamber. Following the sterilization process, the stem explants were cut into 2–3 cm segments with lateral buds (Fig. 2a).

### In vitro culture establishment

**Fig. 3:**
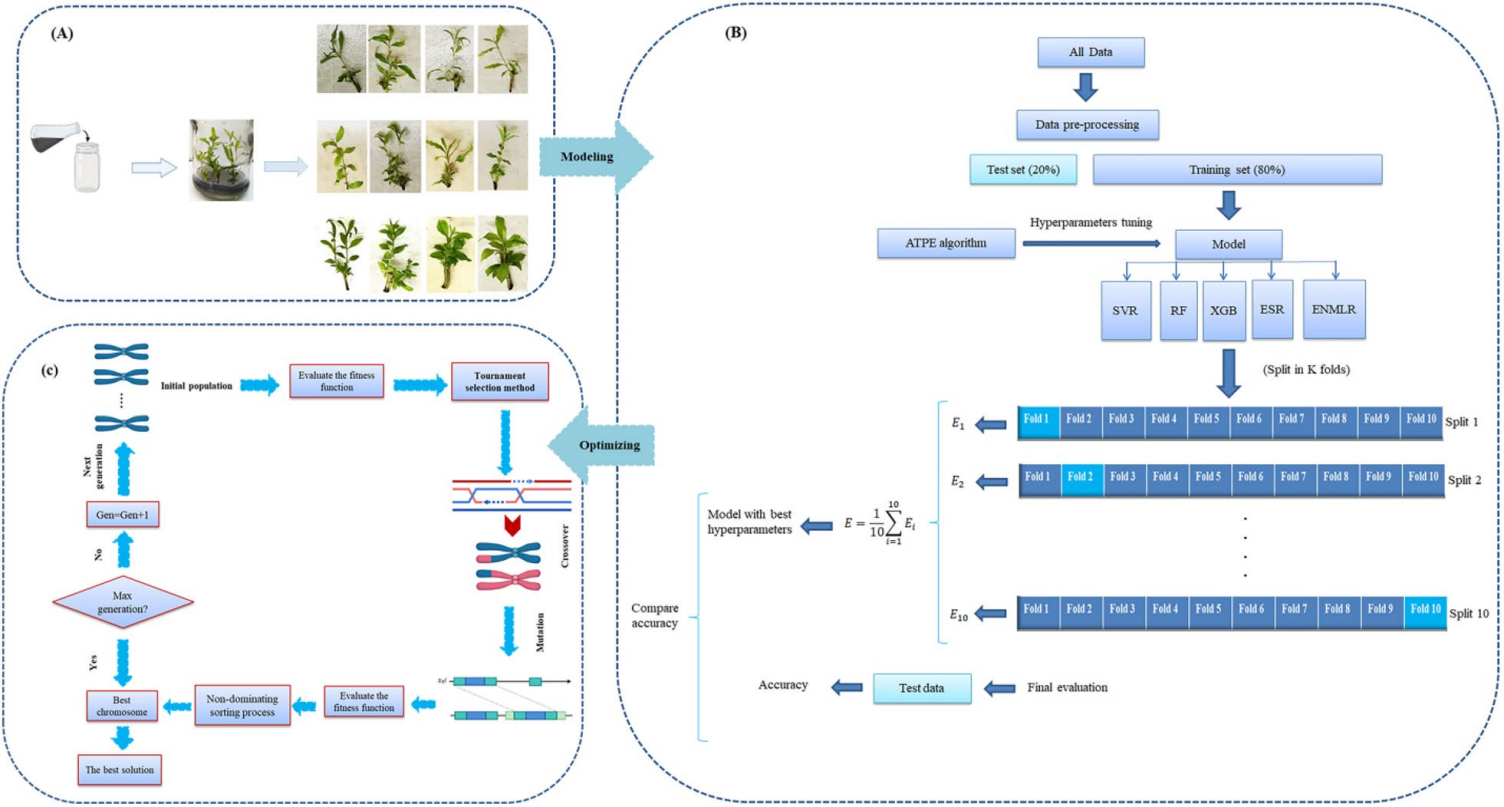
The schematic diagram of the step-by-step procedure of the present research includes (**A**) pomegranate micropropagation, **B** modeling growth parameters based on K-fold cross-validation and ATPE algorithm using MLs, and (**C**) optimization process of growth parameters via non-dominated sorting genetic algorithm-II (NSGA-II)

A preliminary test was carried out using different combinations of culture media: MS (Murashige and Skoog) [34], VS (Van der Salm) [35], WPM (woody plant medium) [36], half-strength MS, and modified MS (mMS), PGRs (BAP and NAA), phenol-controlling compounds (polyvinylpyrolidon, ascorbic acid, and activated charcoal), and silver nitrate (AgNO_3_) as ethylene inhibitor. The main experiment was set up based on the pre-test results, which indicated that the mMS medium supplemented with activated charcoal and AgNO_3_ in combination with either BAP or NAA was the best treatment for stimulating new shoot regeneration. In this experiment, the explants (2–3 cm stem segments with lateral buds) were immediately cultured in the capped glass containers containing 25 mL of mMS as a basal medium supplemented with 1 mg/L BAP, 0.5 mg/L NAA, 250 mg/L activated charcoal, 4.5 mg/L AgNO_3_, 0.7% agar, and 3% sucrose. To obtain the best hormonal composition at the protocol of pomegranate proliferation, the effects of different concentrations of GA_3_ (0, 0.1, 0.25, and 0.5 mg/L) and ZT (0, 0.25, 0.5, and 0.75 mg/L) on shoot proliferation were evaluated. Prior to autoclaving at 121 ℃ for 15 min, the pH of the medium was adjusted to 5.7–5.8. To mitigate tissue culture browning, the cultures were incubated in darkness for 7 days in a growth chamber at a temperature of 25 ± 2 ℃, and then transferred to a 16-h photoperiod with a light intensity of 80 µmol m^−2 ^s^−1^ and an 8-h dark period. After three subcultures on the same culture medium, various morphological responses of the plants were measured for each cultivar; including the proliferation rate (PR; number of new shoots per explant), shoot length (SL; length of new regenerated shoots per explant in cm), leave number (LN; the number of leaves per explant), and explant survival (ES; the survival rate of explants in percent) (Fig. 3a).

### Experimental design and data analysis

The proliferation experiment was carried out using a Completely Randomized Design (CRD) with a factorial arrangement. Each set of treatments consisted of 20 replicates, and subcultures were conducted over a three-week period. The variances analysis was performed using statistical analysis software (version 9.4; SAS Institute, Cary, NC).

### Description of ML models and optimization algorithm

#### Model development

In this study, we employed a range of ML algorithms to build computational models using the datasets as training and testing data. Specifically, we selected most widely used ML algorithms such as SVR, RF, XGB, ENMLR, and ESR to analyze the effect of the independent variables on in vitro pomegranate plant growth responses. These five ML algorithms were applied to different pomegranate cultivars (‘Faroogh’, ‘Atabaki’, and ‘Shirineshahvar’), with two independent variables consisting of various concentrations of GA_3_ and ZT as inputs, and four plant growth responses (PR, SL, LN, and ES) considered as outputs. Prior to applying ML modeling, data scaling was employed to standardize the training set for each cultivar. The features are transformed into a mean of zero and a variance of one by standardizing the data using the Eq. 1. Additionally, Principal Component Analysis (PCA) was used to identify any outlier data; however, no outlier data was found in analysis. To train and test all five models, the experimental data (960 data points) were randomly divided into 80% and 20% for training and testing sets, respectively.

1 $${X}_{std}=\frac{{X}_{o}-\mu }{\sigma }$$

where ${X}_{std}$ is standardized value, ${X}_{o}$ is original value, $\mu$ and $\sigma$ are mean and standard deviation, respectively.

### Hyper parameter optimization in ML models

In ML, the optimization and tuning of hyperparameters in advance play a crucial role in training ML models [37]. These hyperparameters have a significant impact on prediction accuracy and overall performance. Various strategies exist for hyperparameter optimization, including babysitting, grid search, random search, and bayesian optimization [38]. Among these strategies, Bayesian optimization is widely recognized for its generalizability across different test sets and its ability to achieve optimal hyperparameters with fewer iterations. In this study, a novel automatic tuning hyperparameter algorithm called Adaptive Three-structured Parzen Estimator (ATPE) was utilized in Bayesian optimization. This algorithm aimed to adjust the initial hyperparameters of five ML models to achieve optimized performance. It has not yet been applied to the optimization of in vitro PGRs. To improve the generalization performance of these models and avoid overfitting and underfitting, the study combined the ATPE method with K-fold cross-validation (K = 10). By employing the K-fold cross-validation method, all data points were involved in the training phase. The process is illustrated in Fig. 3b. The ML’s hyperparameters and their search space are shown in Table 1. The investigation was conducted with K values ranging from 1 to 10 for K-fold cross-validation. Each K value represented the ATPE algorithm for optimal ML model selection and hyperparameter tuning. One fold was randomly selected as the validation set, while the remaining folds were used to train the model. By employing the K-fold cross-validation method, all data points were involved in the training process.

**Table 1:** Hyperparameter tuning of the constructed models using ATPE

Model	Hyperparameters	Search Space	Type	Description
XGB	n_estimators	[10, 1500]	Integer	The quantity of trees or boosting rounds that need to be constructed. Overfitting may result from larger values
	learning_rate	[0.001, 0.3]	Continues	Shrinking steps in order to avoid overfitting. Lower values could enhance the performance of the model but require more boosting rounds
	gamma	[0, 10]	Continues	Minimum loss reduction to create a new tree split. regulates regularization on nodes in the tree
	max_depth	[1, 20]	Integer	The maximum depth of a tree that restricts the quantity of nodes. aids in managing model complexity
	subsample	[0.001, 1]	Continues	Percentage of training data utilized in each round of boosting. adds randomization to stop overfitting
	colsample_bytree	[0.01, 1]	Continues	Percentage of features utilized in every round of boosting. reduces overfitting by introducing diversity
	min_child_weight	[1, 10]	Continues	The lowest total weight that a child needs in an instance. governs the size of the leaf nodes, affecting the robustness of the model
	reg_lambda	[0, 5]	Continues	L2 regularization term. penalizes big weights to help prevent overfitting
	reg_alpha	[0, 5]	Continues	L1 regularization term. increases the feature matrix's sparsity, which reduces overfitting
RF	n_estimators	[10,1500]	Integer	The number of trees in the forest
	max_features	(0, 1]	Continues	The maximum number of features considered for splitting a node. regulates how diverse each individual trees is in the forest
	max_depth	[2, 50]	Integer	The maximum depth of each tree in the forest. It limits the growth of trees and helps prevent overfitting
	min_samples_split	[1, 100]	Integer	The minimum number of samples needed to divide an internal node. It affects the trees' depth and can stop overfitting
	min_samples_leaf	[1, 15]	Integer	The minimum number of samples needed for a leaf node. It can inhibit overfitting and has an impact on the trees' granularity
	min_weight_fraction_leaf	[0, 0.5]	Continues	The minimum weighted proportion of the total weights necessary for a leaf node to exist. It permits the dataset's occurrences to be weighted differently
	max_leaf_nodes	[2,200]	Integer	The maximum number of leaf nodes in each tree
ENMLR	alpha	[0, 5]	Continues	Determines the strength of regularization. A higher alpha lead to stronger regularization, helping prevent overfitting by penalizing large coefficients
	L1-ratio	[0, 1]	Continues	Controls the balance between L1 and L2 regularization
SVR	Gamma	(0, 10]	Continues	Controls the influence of each training point; higher values lead to a more complex decision boundary
	C	(0, 50]	Continues	Regularization parameter, balancing the trade-off between smooth decision boundary and classifying training points correctly
	epsilon	(0, 5]	Continues	Defines the margin of tolerance for regression errors

*SVR* Support Vector Regression, *RF* Random Forest, *XGB* Extreme Gradient Boosting, *ENMLR* Elastic Net Multivariate Linear Regression, *ESR* Ensemble Stacking Regression

### Support vector regression (SVR)

SVM is a supervised ML method that developed by Vapnik [39]. Initially developed for classification problems (Support Vector Classifier or SVC), SVM was later extended to handle regression problems (SVR) [40]. The fundamental concept behind SVR involves the use of a kernel function to map the original input data into a feature space. The SVM model estimates regression by utilizing a series of kernel functions to convert the original input data from its lower-dimensional representation to a higher-dimensional feature space. Unlike Artificial Neural Network (ANN) models, which often encounter multiple local minima, SVM provides a unique solution results that are at the global optimum. The approximated function within the SVR algorithm can be expressed as follows:

2 $$f\left(x\right)={\omega }^{T}x+b with \omega \epsilon x, b\epsilon R$$

where $f\left(x\right)$ represents the estimated output value, $\omega$ denotes weight for the ${\text{i}}^{\text{th}}$ sample point, and $b$ represents the bias. The values of $\omega$ and $b$ are determined by minimizing the regularized risk function, which is expressed as:

3 $$R(C)=C\frac{1}{n}\sum_{i=1}^{n}L\left({d}_{i},{y}_{i}\right)+\frac{1}{2}{\Vert \omega \Vert }^{2}$$

where $C$ represents the penalty parameter that balances the trade-off between model complexity and training error, ${d}_{i}$ denotes the desired value, $n$ represents the total number of observations, and $C\frac{1}{n}\sum_{i=1}^{n}L\left({d}_{i},{y}_{i}\right)$ is the empirical error. The following equation is employed to determine the insensitive loss function (${l}_{\varepsilon })$:

4 $${l}_{\varepsilon }\left(d,y\right)=\left|d-y\right|-\varepsilon \left|d-y\right|\ge \varepsilon or 0 otherwise$$

where $\frac{1}{2}{\Vert \omega \Vert }^{2}$ represents the regularization term, while ɛ (epsilon) represents the insensitive tube. The approximated function in Eq. (2) can be explicitly expressed by incorporating Lagrange multipliers and leveraging the optimality constraints. By introducing the Lagrange multipliers $({a}_{i})$, the function is given by:

5 $$f\left(x,{a}_{i},{a}_{i}^{*}\right)=\sum_{i=1}^{n}\left({a}_{i}-{a}_{i}^{*}\right)K({x}_{i},{x}_{i}^{T})+b$$

where $K({x}_{i},{x}_{i}^{T})$ represents the kernel function. The Radial Basis Function (RBF) non-linear kernel function plays a crucial role in mapping of input vectors nonlinearly into a high-dimensional feature space. In this study, the RBF was utilized due to its superior performance in estimating the H estimations compared to other kernel functions.

6 $${K}_{rbf}({x}_{i},{x}_{i}^{T})=\text{exp}\left[\frac{{-\left({x}_{i}-{x}_{i}^{T}\right)}^{2}}{{2\sigma }^{2}}\right]$$

### Random forest (RF)

RF introduced for classification or regression prediction algorithm introduced by Breiman [41]. It solves the performance limitations of decision trees and exhibits favorable characteristics such as robustness to noise and outliers, scalability, and parallelism in high-dimensional data classification tasks. RF overcomes the "dimensionality disaster" often encountered in big data scenarios that often other models fail to perform effectively. Additionally, RF demonstrates comparable error rates to other methods across various learning tasks and exhibits a reduced tendency to overfitting. Notably, RF is a well-known bagging algorithm that excels in regression problems [38]. RF algorithm combines decision tree-based techniques with ensemble methods, effectively leveraging their synergistic benefits, making it a suitable choice as one of the foundational models in the ensemble model employed in this study. The formula of RF is as follows:

7 $${\text{i}}\widehat{\text{y}}\left({\text{x}}_{\text{i}}\right)\text{=}\frac{1}{{\text{K}}}\sum_{k=1}^{K}{\text{T}}_{{\text{D}}\left({\theta }_{\text{k}}{}\right)}\left({\text{x}}_{\text{i}}\right), k =\{1, 2, \dots ,K\}$$

where ${\text{x}}_{\text{i}}$ refers to the value of the sample proportion, ${\text{D}}\left({\theta }_{\text{k}}\right)$ denotes a different bootstrapped sample, and ${\text{K}}$ is tree number (${\text{T}}_{{\text{D}}\left({\theta }_{K}\right)}\text{)}$.

### eXtreme Gradient Boosting (XGB)

XGB is an advanced supervised learning algorithm proposed by Chen and Guestrin [42]. This method is based on the Gradient-Boosted Decision Tree (GBDT) approach. XGB aims to create a “strong” learner by combining predictions from a collection of “weak” learners using additive training strategies. This algorithm incorporates a second-order Taylor expansion of the loss function and a regular term, which effectively mitigates overfitting and expedites convergence. The XGB algorithm enhances prediction accuracy by iteratively constructing new decision trees with continuously diminish the residuals between predicted and observed values. XGB stands out as a prominent open-source boosting tree toolkit, offering remarkable speed and performance advantages over other gradient-boosting methods. It is more than 10 times faster than common toolkits, making it the preferred selection for massively parallel boosting tree tasks. XGB prediction for *i* instance is:

8 $${f}_{i}^{(d)}=\sum_{k=1}^{d}{f}_{k}({x}_{i})={f}_{i}^{(d-1)}{f}_{d}{(x}_{i})$$

where ${f}_{k}({x}_{i})$ represents the learner at step $d$, the predictions at steps $d$ and $d-1$ are denoted as ${f}_{i}^{(d)}$ and ${f}_{i}^{(d-1)}$, respectively and ${x}_{i}$ represents the input variable.

In order to prevent the problem of overfitting without sacrificing the computational speed of the model, XGB employs an analytical expression to evaluate the “goodness” of the model in relation to the original function. This analytical formula, denoted as Eq. (2), is created by XGB to provide an estimate of the model’s “goodness” while also reducing the computational speed associated with mathematical computations.

9 $${\text{Objective}}^{(d)}=\sum_{k=1}^{n}l\left({\overline{y} }_{i},{y}_{i}\right)+\sum_{k=1}^{d}\sigma {(f}_{i})$$

where $l$ is the loss function, $n$ indicates the observation number used, and $\sigma$ denotes the regularization term as represented in Eq. (3).

10 $$\sigma \left(f\right)=\gamma T+0.5\lambda {\Vert \omega \Vert }^{2}$$

where $\omega$ denote the vector of scores associated with leaves, $\lambda$ represents the regularization parameter, and $\gamma$ indicates the minimum loss required for further partitioning of a leaf node.

### Elastic net multivariate linear regression (ENMLR)

ENMLR is a regression technique that combines two effective shrinkage regression methods: Ridge regression (L2 penalty) and LASSO regression (L1 penalty). Ridge regression is employed to address high-multicollinearity problems, while LASSO regression focuses on feature selection in regression coefficients. The elastic net estimator in ENMLR benefits from ridge regularization, which allows for better handling of correlations between predictors compared to LASSO regression. Simultaneously, the L1 regularization in elastic net promotes sparsity, facilitating the identification of essential features. However, similar to LASSO regression, the bias issue is still present in ENMLR. The elastic net estimator minimizes the following expression:

11 $$EN \left(\beta \right)=\sum_{i=1}^{n}{\left({y}_{i}-{x}_{i}^{T}\beta \right)}^{2}+{\lambda }_{1} \sum_{j=1}^{p}\left|{\beta }_{j}\right|+{\lambda }_{2}\sum_{j=1}^{p}{\left|{\beta }_{j}\right|}^{2}$$

where $\beta$ is the regression coefficients, ${\beta }_{j}$ is the regression coefficient of the ${j}^{th}$ predictor variable, ${\lambda }_{1}$ and ${\lambda }_{2}$ are the tuning parameters coming from Lasso and Ridge, respectively and positive numeric values (${\lambda }_{1}$, ${\lambda }_{2}$> 0). λ is a penalty parameter and has the effect of a compression variable, and its numerical value indicates the severity of punishment.

### Ensemble stacking regression (ESR)

The stacking regressor, initially introduced by Wolpert [43], is an effective ensemble learning technique that combines multiple regression models to improve prediction accuracy. In this approach, a meta-regressor is trained to aggregate the predictions of the base regressors, thereby leveraging the collective knowledge of the individual models Li et al. [44]. Different techniques, such as stacking, weighted averaging, and direct averaging, can be employed to create ensemble regressors by integrating the predictions of the base models [45]. The choice of the specific technique depends on finding an optimal balance for combining the predictions, and the meta-regressor can be any type of regression models [46]. To implement stacking regression, the new meta feature sets generated by each base regressor are merged to form the meta training set, and the new target sets produced by each base regressor are combined to create the meta testing set. The final predictions are then generated by the meta-regressor, which is trained using the new meta training set Wu et al. [25]. The stacking regression methodology has gained popularity in various domains, including molecular quantum characteristics [44], daily reference evapotranspiration estimation [25], genome prediction [47], and stock portfolio prediction [48]. In this particular study, XGB, SVR, and ENMLR models were utilized as the base regressors, while RF was employed as the meta-regressor.

### Performance evaluation

In order to evaluate and compare the accuracy and performance of the developed ML algorithms in predicting the proliferation of pomegranate, five popular statistical quantitative indicators, namely the correlation coefficient (R), Coefficient of Determination (R^2^), Root Mean Square Error (RMSE), Relative Root Mean Squared Error (RRMSE), Mean Absolute Error (MAE), and Mean Absolute Percentage Error (MAPE), were utilized. These quantitative indicators can be found in Table 2.

**Table 2:** Description of statistical indicators for the constructed models evaluation

Performance criteria	Formula	Description
Correlation coefficient	$R=\frac{\sum_{i=1}^{n}\left({O}_{i}-\overline{O }\right)\left({P}_{i}-\overline{P }\right)}{\sqrt{\sum_{i=1}^{n}{\left({O}_{i}-\overline{O }\right)}^{2}}\sqrt{\sum_{i=1}^{n}{\left({P}_{i}-\overline{P }\right)}^{2}}}$	R is a statistical measure that quantifies the degree of correlation between observed and predicted values. The model's predictability improves as it approaches 1
Coefficient of determination (7)	${R}^{2}={\left(\frac{\sum_{i=1}^{n}\left({O}_{i}-\overline{O }\right)\left({P}_{i}-\overline{P }\right)}{\sqrt{\sum_{i=1}^{n}{\left({O}_{i}-\overline{O }\right)}^{2}{\left({P}_{i}-\overline{P }\right)}^{2}}}\right)}^{2}$	R^2^ represents the proportion of the variance in the observed data that is explained by the regression model. As R^2^ approaches 1, the model’s ability to account for the variability in the data improves
Root mean squared error	$RMSE={\left(\frac{1}{n}\sum_{i=1}^{n}{\left({O}_{i}-{P}_{i}\right)}^{2}\right)}^{0.5}$	RMSE used to describe the average squared difference between the measured and predicted values. It determines the concentration of data around the optimal fit line The lower, the better model’s performance
Mean absolute error	$MAE=\frac{1}{n}\sum_{i=1}^{n}\left|{O}_{i}-{P}_{i}\right|$	MAE used to describe the sum of the absolute differences between the measured and predicted values. It does not punish high mistakes that result from outliers. It furthermore offers a reliable gauge of the model's accuracy The lower, the better model’s performance
Relative root mean squared error	$RRMSE(\%)=\frac{100}{\overline{O} }{\left(\frac{1}{n}\sum_{i=1}^{n}{\left({O}_{i}-{P}_{i}\right)}^{2}\right)}^{0.5}$	RRMSE is the RMSE, which normalized by mean of observations model performance is considered as: excellent for $RRMSE<$ 10% good for $10\%<RRMSE<20\%$ fair for $20\%<RRMSE<30\%$ poor for $RRMSE>30\%$
Mean absolute percentage error	$MAPE(\%)=\frac{100}{n}\sum_{i=1}^{n}\left(\frac{\left|{O}_{i}-{P}_{i}\right|}{{P}_{i}}\right)$	MAPE used to describe the absolute inaccuracy of the observed and predicted variables in percentage model performance is considered as: excellent for MAP $E<$ 10% good for $10\%<MAPE<20\%$ acceptable for $20\%<MAPE<50\%$ inaccurate for MAP $E>50\%$

Where $n$ is total measurement, ${O}_{i}$ and ${P}_{i}$, are observed and predicted values, $\overline{O }$ and $\overline{P }$ stand for mean of observed and predicted values, respectively

### Global performance *indicator* (GPI)

In order to enhance the accuracy and reliability of statistical analysis and to mitigate any potential discrepancies, we employed the GPI method. Despotovic et al. [49] were the pioneers in introducing GPI as a novel aspect. GPI is a remarkable technique that combines the effects of multiple statistical indicators. During the process, all statistical indicators are scaled to a range between 0 and 1. Subsequently, the appropriate median value of all models is subtracted from each scaled value of a statistical indicator. These differences are then aggregated using appropriate weighting factors (a weight of -1 for $R$ and ${R}^{2}$ and a weight of 1 for all other statistical indicators). The model with higher GPI values is considered the best. The following equation represents the GPI model:

12 $${GPI}_{i}=\sum_{j=1}^{5}{\alpha }_{j}\left({\text{\rm M}}_{j}^{S}-{\text{\rm I}}_{ij}^{S}\right)$$

where ${GPI}_{i}$ represents global performance indicator for model $i$, ${\text{\rm M}}_{j}^{S}$ is median of scaled values of indicator $j$, ${\text{\rm I}}_{ij}^{S}$ is the scaled value of indicator $j$ for model $i$, ${\alpha }_{j}$ equals -1 for both $R$ and ${R}^{2}$ and 1 for other performance criteria.

### Optimization of ML model via non‑dominated sorting genetic algorithm‑II (NSGA-II)

The best ML algorithm as the fitness function was introduced to the Non-dominating Sorting Genetic Algorithm (NSGA-II) as optimization algorithm in order to find the optimal combination of inputs (GA_3_ and ZT) for achieving maximal growth responses in three cultivars (Fig. 3c). Based on natural selection, this study employed several parameters to ensure the effectiveness of the NSGA-II optimization process. The first step in the NSGA-II process involved the creation of an initial population, where all the chromosomes were constructed. Then the tournament selection method was adopted to select an elite population for crossover. A binary crossover function, a well-known crossover technique, was considered to generate the next generation of chromosomes. To introduce diversity into the population and prevent convergence to local optima, a mutation operator was applied. It introduced random variations into the chromosomes, reducing the possibility of having similar chromosomes within the population [50]. The non-dominated sorting concept was utilized to derive non-dominated solutions, with each non-dominated front assigned a rank or level date. The non-dominated front with the highest rank is removed, and the remaining solutions were used to generate the parent population for the next generation. Crowding distance was employed to estimate the objective function, and solutions categorized by crowding distance in descending order based on the lowest density of solutions with less priority. In order to achieve an improved fitness function during the optimization process, the optimal values for crucial operators such as the crossover rate, maximum generation, initial population, and mutation rate were regulated through trial and error. In the current study, the crossover rate was set at 90% with a distribution index of 15, the maximum generation was set to 200, the initial population size was 100, and a distribution index of 20 was used for the mutation operator which was real-valued polynomial mutation (real_pm) (Fig. 3c).

All mathematical codes for implementing and evaluating ESR, RF, SVR, and ENMLR models were performed using the Python library Scikit-learn version 1.3.2 [51]. Additionally, XGB was performed using the XGBoost library version 2.0.3 [42]. The tuning of hyperparameters for each of the five models (SVR, RF, XGB, ESR, and ENMLR) was conducted using the Hyperopt library version 0.2.7 [52], and the Pymoo library version 0.6.1.1 [53], specifically applied for multi-objective optimization (NSGA-II algorithm).

## Results

### The effect of PGRs on in vitro shoot proliferation and development of pomegranate

According to data analysis using factorial ANOVA, the growth responses of pomegranate, including LN, PR, ES, and SL were found to be significantly influenced by different concentrations and combinations of PGRs (GA_3_ and ZT), as well as the cultivar type. The detailed results can be found in Table 3.

**Table 3:** Effect of different concentrations of PGRs on in vitro growth parameters of pomegranate cultivars

Cultivar	PGRs (mg/L)		LN	PR	ES (%)	SL (cm)
	GA_3_	ZT				
‘Atabaki’	0	0	15.75 ± 0.775^yz^	2.69 ± 0.479^k−l^	77.50 ± 2.582^g^	1.72 ± 0.075^t−v^
	0	0.25	16.44 ± 0.814^u−x^	3.19 ± 0.403^f−i^	78.44 ± 2.394^fg^	1.86 ± 0.143^t−r^
	0	0.50	16.56 ± 0.814^s−x^	3 ± 0.365^i−l^	81.56 ± 7.465^d−f^	2.09 ± 0.128^op^
	0	0.75	16.94 ± 0.854^q−u^	3.12 ± 0.341^g−j^	82.5 ± 6.831^c−e^	2.21 ± 0.112^no^
	0.1	0	16.06 ± 1.181^w−z^	2.69 ± 0.479^l−n^	77.5 ± 2.582^g^	1.73 ± 0.141^tu^
	0.1	0.25	16.50 ± 1.033^t−x^	3.06 ± 0.574^h−k^	78.75 ± 2.236^e−g^	2.05 ± 0.187^o−q^
	0.1	0.50	16.87 ± 0.885^q−u^	3.12 ± 0.342^g−j^	83.12 ± 8.539^cd^	2.37 ± 0.057^mn^
	0.1	0.75	17.19 ± 0.911^p−s^	3.19 ± 0.544^f−i^	85 ± 8.944^b−d^	2.51 ± 0.043^lm^
	0.25	0	15.44 ± 1.153^z^	2.62 ± 0.500^mn^	78.12 ± 2.500^fg^	2.00 ± 0.229^p−r^
	0.25	0.25	18.06 ± 1.181^j−n^	2.81 ± 0.403^j−n^	82.50 ± 6.831^c−e^	2.62 ± 0.155^kl^
	0.25	0.50	17.12 ± 0.885^p−t^	2.87 ± 0.342^i−n^	83.75 ± 8.062^cd^	2.78 ± 0.113^i−k^
	0.25	0.75	17.12 ± 0.957^p−t^	3.19 ± 0.403^f−i^	86.25 ± 9.574^bc^	2.90 ± 0.125^h−j^
	0.50	0	16.06 ± 1.181^w−z^	2.94 ± 0.680^i−m^	77.19 ± 2.562^g^	2.97 ± 0.388^h^
	0.50	0.25	16.75 ± 0.775^r−v^	3.12 ± 0.342^g−j^	**100 ± 0.000**^**a**^	4.05 ± 0.616^d^
	0.50	0.50	16.87 ± 1.025^q−u^	**3.56 ± 0.512**^**b−e**^	**100 ± 0.000**^**a**^	3.99 ± 0.532^d^
	0.50	0.75	**18.25 ± 1.183**^**i−m**^	**3.56 ± 0.512**^**b−e**^	**100 ± 0.000**^**a**^	**6.75 ± 0.491**^**a**^
‘Faroogh’	0	0	19.62 ± 0.619^de^	3.56 ± 0.629^b−e^	82.50 ± 5.773^c−e^	2.52 ± 0.192^lm^
	0	0.25	19.62 ± 0.619^de^	3.75 ± 0.447^a−d^	83.12 ± 4.787^cd^	2.85 ± 0.113^h−j^
	0	0.50	19.62 ± 0.619^de^	3.81 ± 0.403^a−c^	83.12 ± 4.787^cd^	2.78 ± 0.127^i−k^
	0	0.75	19.69 ± 0.479^de^	3.81 ± 0.403^a−c^	83.75 ± 5.000^cd^	2.75 ± 0.169^jk^
	0.1	0	19.56 ± 0.629^d−f^	3.75 ± 0.447^a−d^	83.75 ± 5.000^cd^	2.79 ± 0.196^ij^
	0.1	0.25	19.62 ± 0.619^de^	3.75 ± 0.447^a−d^	86.25 ± 6.191^bc^	2.92 ± 0.135^hi^
	0.1	0.50	19.94 ± 0.772^d^	3.81 ± 0.403^a−c^	84.37 ± 5.123^cd^	2.86 ± 0.135^h−j^
	0.1	0.75	19.62 ± 0.619^de^	3.81 ± 0.403^a−c^	83.75 ± 5.000^cd^	2.79 ± 0.196^ij^
	0.25	0	19.69 ± 0.602^de^	3.75 ± 0.447^a−d^	83.75 ± 5.000^cd^	2.84 ± 0.143^h−j^
	0.25	0.25	19.19 ± 0.834^e−g^	3.81 ± 0.403^a−d^	83.75 ± 5.000^cd^	3.15 ± 0.125^g^
	0.25	0.50	20.06 ± 0.772^d^	3.87 ± 0.341^ab^	83.75 ± 5.000^cd^	3.36 ± 0.124^f^
	0.25	0.75	20 ± 0.730^d^	3.87 ± 0.342^ab^	88.75 ± 7.188^b^	3.54 ± 0.082^e^
	0.50	0	19.75 ± 0.447^de^	3.69 ± 0.479^a−e^	84.37 ± 5.124^cd^	2.80 ± 0.087^ij^
	0.50	0.25	22.06 ± 1.569^c^	3.69 ± 0.479^a−e^	**100 ± 0.000**^**a**^	4.40 ± 0.647^c^
	0.50	0.50	22.94 ± 1.769^b^	3.75 ± 0.447^a−d^	**100 ± 0.000**^**a**^	4.55 ± 0.544^bc^
	0.50	0.75	**23.62 ± 1.670**^**a**^	**4.00 ± 0.000**^**a**^	**100 ± 0.000**^**a**^	**4.62 ± 0.506**^**b**^
‘Shirineshahvar’	0	0	11.31 ± 1.138^A^	2.31 ± 0.704^m^	43.12 ± 4.787^p^	1.26 ± 0.169^z^
	0	0.25	15.94 ± 0.929^x−z^	3.12 ± 0.342^g−j^	47.50 ± 5.773^o^	1.41 ± 0.063^yz^
	0	0.50	17.37 ± 0.619^o−r^	3.5 ± 0.516^c−f^	50 ± 6.324^l−o^	1.54 ± 0.069^w−y^
	0	0.75	18.37 ± 0.619^h−m^	3.44 ± 0.512^d−g^	51.87 ± 4.031^k−n^	1.55 ± 0.079^v−y^
	0.1	0	16.12 ± 1.025^v−y^	3.44 ± 0.512^d−g^	48.75 ± 6.191^no^	1.42 ± 0.082^yz^
	0.1	0.25	17.75 ± 0.774^m−p^	3.44 ± 0.512^d−g^	52.50 ± 4.472^k−n^	1.51 ± 0.051^xy^
	0.1	0.50	18.00 ± 0.816^k−o^	3.50 ± 0.516^c−f^	52.50 ± 4.472^k−n^	1.64 ± 0.040^u−x^
	0.1	0.75	18.69 ± 0.793^g−j^	3.50 ± 0.516^c−f^	53.12 ± 4.787^j−m^	1.69 ± 0.036^u−w^
	0.25	0	16.69 ± 1.138^s−w^	3.44 ± 0.512^d−g^	49.37 ± 6.801^m−o^	1.52 ± 0.055^xy^
	0.25	0.25	17.94 ± 0.772^l−o^	3.44 ± 0.512^d−g^	53.75 ± 6.191^j−l^	1.71 ± 0.058^t−v^
	0.25	0.50	18.19 ± 0.655^j−m^	3.56 ± 0.512^b−e^	55.00 ± 5.164^jk^	1.79 ± 0.075^s−u^
	0.25	0.75	18.87 ± 0.806^g−i^	3.56 ± 0.512^b−e^	55.00 ± 5.164^jk^	1.90 ± 0.049^s−r^
	0.50	0	17.44 ± 1.093^n−q^	3.37 ± 0.500^e−h^	46.87 ± 4.787^op^	1.53 ± 0.058^w−y^
	0.50	0.25	18.62 ± 0.957^g−k^	3.50 ± 0.516^c−f^	56.87 ± 7.932^ij^	1.79 ± 0.129^s−u^
	0.50	0.50	18.50 ± 1.155^h−l^	3.50 ± 0.516^c−f^	59.37 ± 6.801^hi^	**1.95 ± 0.078**^**s−r**^
	0.50	0.75	**18.94 ± 0.772**^**f−h**^	**3.56 ± 0.512**^**b−e**^	**61.87 ± 7.500**^**h**^	1.91 ± 0.093^s−r^

The results were expressed as the mean ± standard deviation (n = 20)

*GA*_*3*_ gibberellic acid, *ZT* zeatin, *PR* proliferation rate, *SL* shoot length, *LN* leave number, and *ES* explant survival

Bold values have mentioned the biggest and best values, respectively.

The addition of ZT to the growth medium, particularly at a concentration of 0.75 mg/L, resulted in improved shoot regeneration favorable vegetative growth characteristics per explant when compared to the control medium. Based on the results of Table 3, although the positive changes in the growth parameters were primarily attributed to increasing the concentrations of PGRs and the interaction between them, the combination of the highest concentration of ZT and GA_3_ treatment was the most effective treatment in promoting overall growth response. Specifically, when the media was augmented with 0.50 mg/L GA_3_ and 0.75 mg/L ZT the average growth response was significantly enhanced (Table 3). It is important to note that the observed changes in the growth parameters were different based on the cultivar type. Among the three cultivars studied, the ‘Faroogh’ cultivar exhibited the maximum values of LN (23.62), and PR (4). Similarly, the ‘Atabaki’ cultivar showed the highest growth responses in SL (6.75 cm) when treated with 0.50 mg/L GA_3_ and 0.75 mg/L ZT. Regarding ES, both ‘Faroogh’ and ‘Atabaki’ cultivars demonstrated a maximum value of ES which was 100% when exposed to three treatments involving the interaction of 0.25, 0.50, 0.75 mg/L ZT with 0.50 mg/L GA_3_. In contrast, the ‘Shirineshahvar’ cultivar exhibited lower ES rates than other cultivars. For this particular cultivar, the same treatment interaction as mentioned earlier led to the highest values of LN (18.94), PR (3.56), ES (61.87%), and SL (1.95 cm). Generally, the highest and lowest overall growth responses were achieved in the ‘Faroogh’ and ‘Shirineshahvar’, respectively (Table 3).

### Comparison of ML performance

**Fig. 4:**
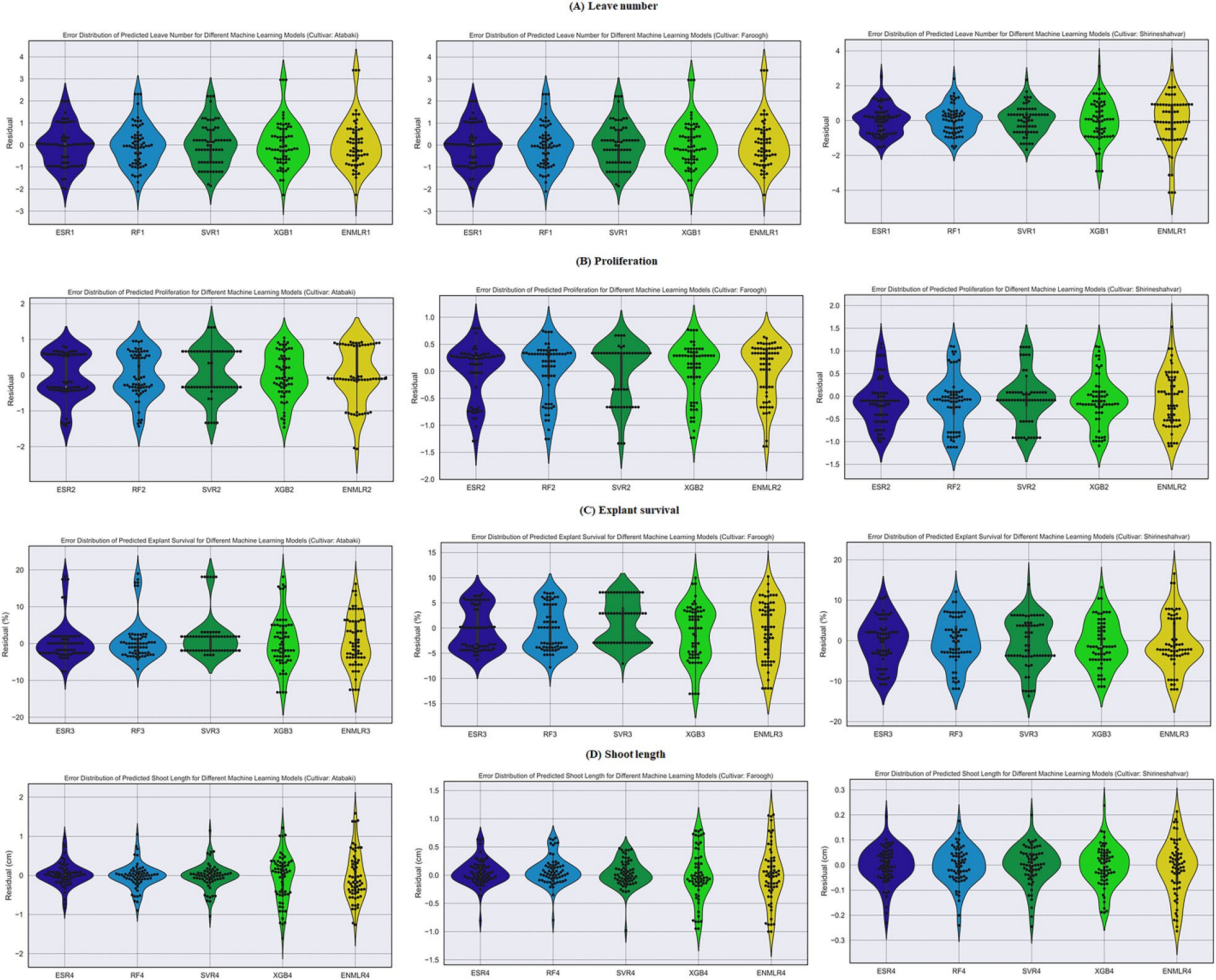
The violin plots of the performance metrics of analyzed models on the observed value vs. the predicted values on in vitro pomegranate growth parameters including: **A** leave number, **B** proliferation, **C** explant survival, **D** shoot length

**Fig. 5:**
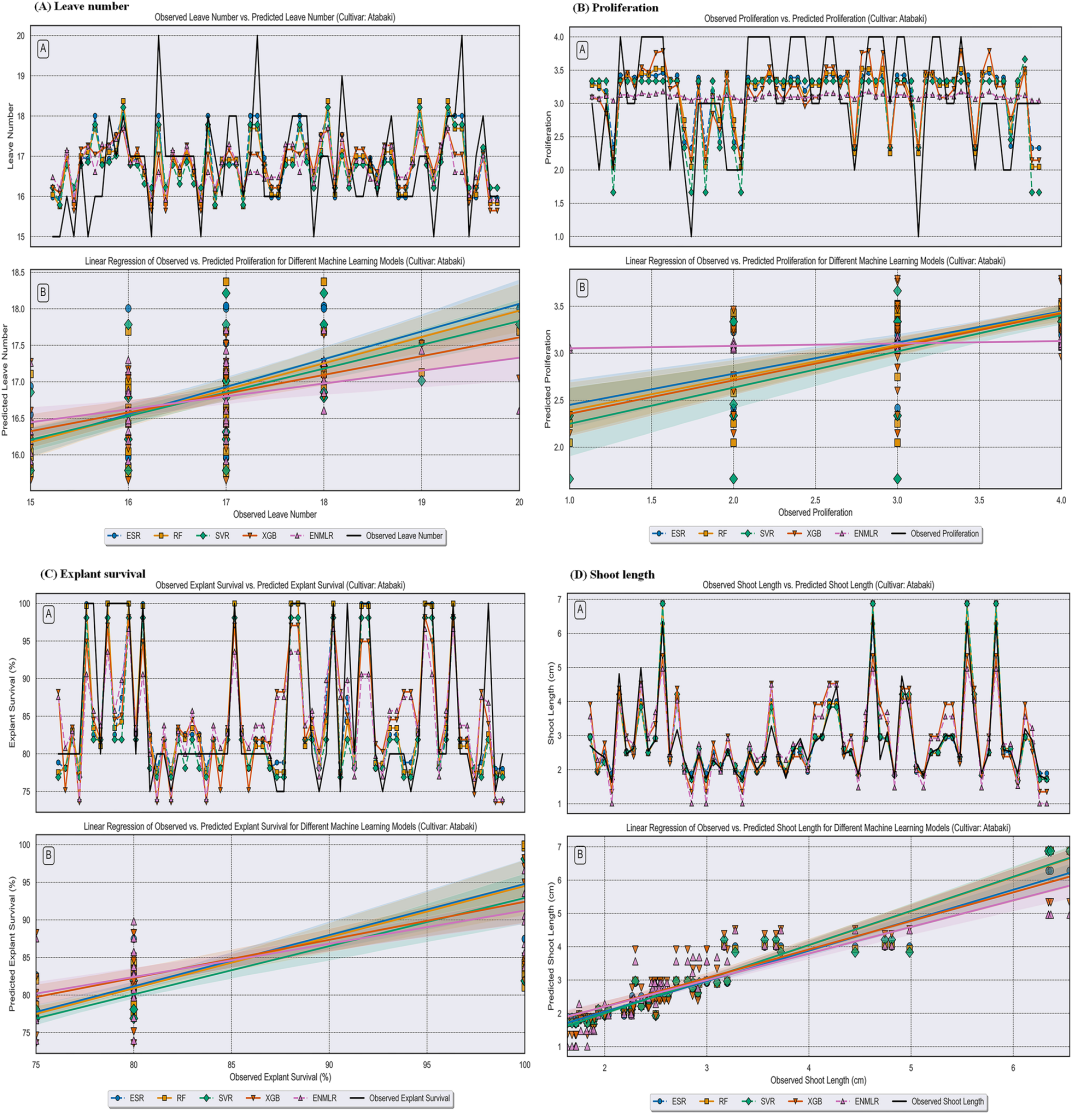
Comparison between the predicted compressive strength via RF, XGB, SVR, ESR, and ENMLR models. **A** leave number, **B** proliferation, **C** explant survival, **D** shoot length of the pomegranate cultivar ‘Atabaki’

**Fig. 6:**
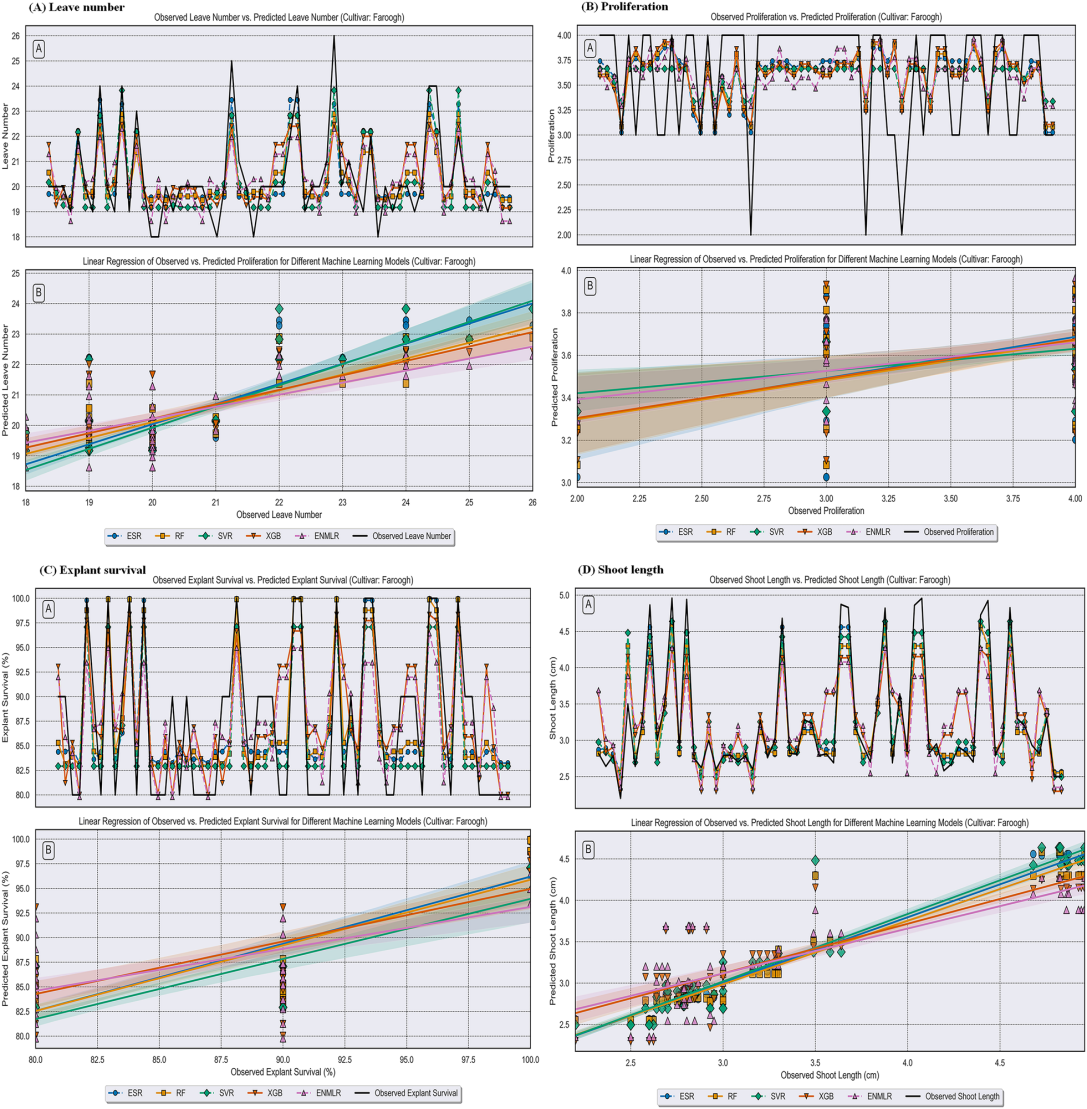
Comparison between the predicted compressive strength via RF, XGB, SVR, ESR, and ENMLR models. **A** leave number, **B** proliferation, **C** explant survival, **D** shoot length of the pomegranate cultivar ‘Faroogh’

**Fig. 7:**
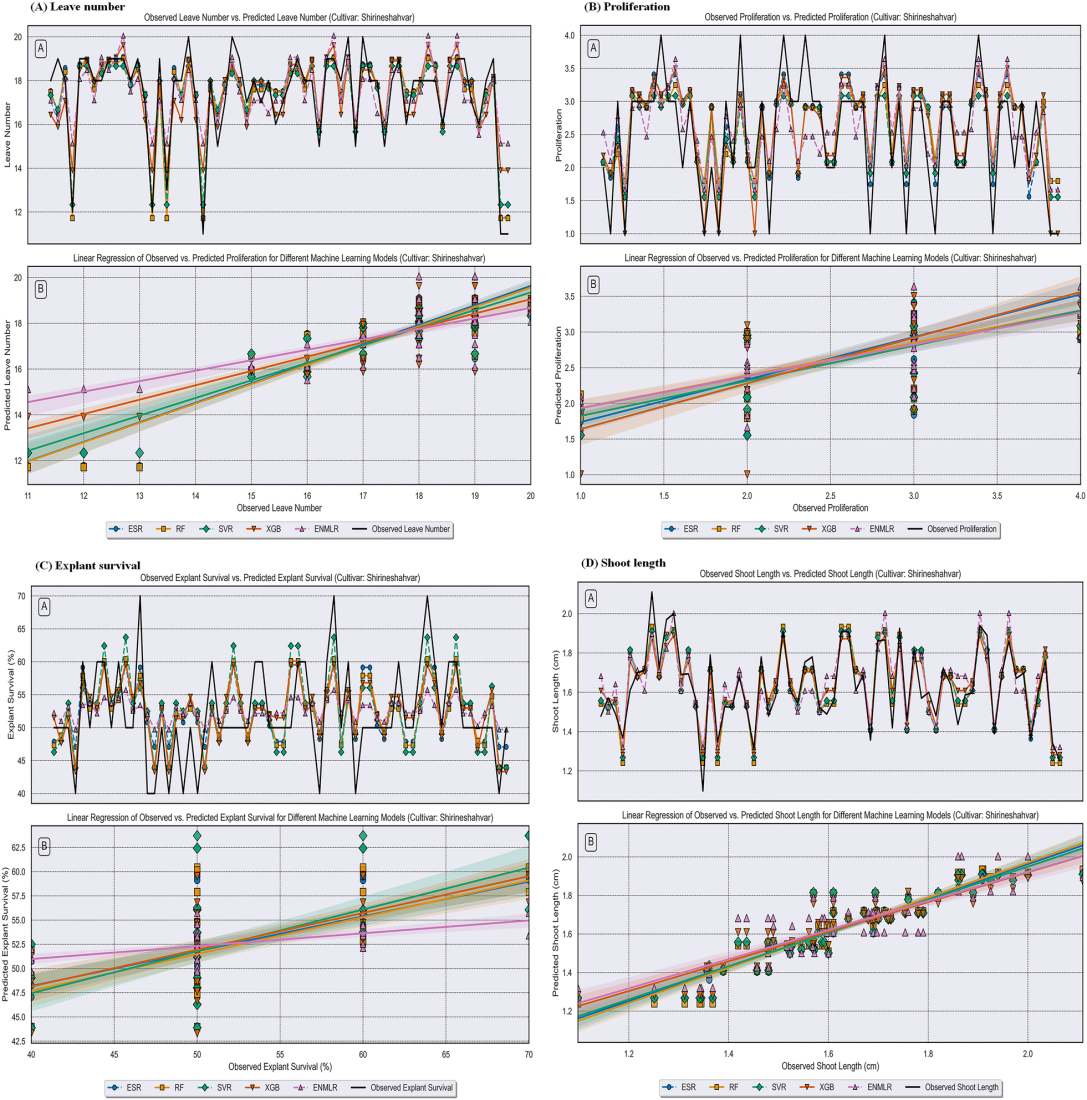
Comparison between the predicted compressive strength via RF, XGB, SVR, ESR, and ENMLR models. **A** leave number, **B** proliferation, **C** explant survival, **D** shoot length of the pomegranate cultivar ‘Shirineshahvar’

In the present study, we utilized the advantages of five ML algorithms namely RF, XGB, SVR, ESR, and ENMLR to build the mathematical models. The scatter plots in Figs. 5, 6 and 7 illustrate the prediction results of these models, while the corresponding prediction evaluation indexes are shown in Tables 4, 5, and 6. Violin plots of the performance metrics are presented in Fig. 4. When comparing the ENMLR to other ML algorithms for all parameters (outputs), both the training and test subset R-values, which measure the correlation between observed (experimental) and predicted values of ML algorithms, were lower. This indicates that all five ML models had a good performance and predictability. However, the ESR with higher R and R^2^ and smaller RRMSE, RMSE, MAE, and MAPE values in both training and testing sets was the best algorithm in comparison to four other models for all growth parameters (Tables 4, 5 and 6). In this regard, the results derived by comparing the statistical indicators of the different models on the measured growth parameters revealed that the values of the ESR was very close to the other ML algorithms in all three cultivars. Moreover, the impact of statistical quantitative indicators was not clearly distinguishable and different statistical indicator values are in favor for different models; therefore, to address this vagueness, the GPI for the test dataset of overall ML logarithms was calculated and presented in Table 7. The GPI estimation ranked the ESR model as the top performer among all other models. Calculated GPI revealed the order of ESR vs. XGB, RF, SVR, and ENMLR models were: 1.829 vs. − 1.674, 0.647, 0, − 4.171, for LN of ‘Atabaki’ cultivar; 1.312 vs. − 2.562, 0, 0.525, and − 4.688 for LN of ‘Faroogh’ cultivar; 0.089, − 3.040, 0.032, 0.004, and − 5.911 for LN of ‘Shirineshahvar’ cultivar; 1.383 vs 0.980, 0.738, − 2.326, and − 3.801, for PR of ‘Atabaki’ cultivar; 1.182 vs. − 1.199, 0.567, − 2.121, and − 2.104 for PR of ‘Faroogh’ cultivar; 1.911, 0.574, − 2.616, 0.255, and − 3.807 for PR of ‘Shirineshahvar’ cultivar; 0.933 vs. − 4.870, 0.573, 0, − 4.814, for ES of ‘Atabaki’ cultivar; 0.748 vs. − 3.813, 0.483, 0.085, and − 5.240 for ES of ‘Faroogh’ cultivar; 0.973, 0.818, − 1.501, − 2.966, and − 2.507 for ES of ‘Shirineshahvar’ cultivar; 0.180 vs. − 5.158, 0.108, 0.035, − 5.782, for SL of ‘Atabaki’ cultivar; 0.619 vs. − 4.058, 0.092, 0.405, and − 5.380 for SL of ‘Faroogh’ cultivar; 0.513, − 0.913, 0.193, 0.150, and − 5.487 for SL of ‘Shirineshahvar’ cultivar (Table 7). Additionally, the regression lines demonstrated the good fit correlation between the observed and predicted data for all growth parameters during both the training and testing phases of the ML models (Figs. 5, 6, and 7).

**Table 4:** Statistical evaluation of the constructed models for the micropropagation of the pomegranate cultivar ‘Atabaki’

Cultivar	Model	Study parameter	Data sets			Performance criteria				
				RRMSE (%)	RMSE		MAE	MAPE (%)	R^2^	R
‘Atabaki’	XGB	LN	Trainng Set	6.07	1.018		0.817	4.92	0.356	0.597
	RF			5.67	0.952		0.774	4.66	0.350	0.592
	SVR			5.77	0.969		0.803	4.83	0.333	0.577
	ENMLR			6.21	1.042		0.836	5.03	0.222	0.471
	ESR			5.82	0.976		0.770	4.65	0.317	0.563
	XGB		Testing Set	6.06	1.019		0.781	4.59	0.296	0.544
	RF			5.67	0.953		0.728	4.30	0.381	0.617
	SVR			5.70	0.958		0.761	4.51	0.381	0.617
	ENMLR			6.50	1.094		0.820	4.81	0.185	0.430
	ESR			5.48	0.922		0.698	4.15	0.425	0.652
	XGB	PR	Trainng Set	20.42	0.634		0.526	20.98	0.254	0.504
	RF			19.51	0.606		0.523	20.74	0.421	0.649
	SVR			19.56	0.607		0.549	21.05	0.412	0.642
	ENMLR			24.87	0.772		0.574	25.09	0.264	0.514
	ESR			19.88	0.617		0.552	21.95	0.394	0.628
	XGB		Testing Set	20.43	0.635		0.528	20.56	0.358	0.598
	RF			20.60	0.640		0.555	21.27	0.347	0.589
	SVR			21.73	0.675		0.596	22.08	0.300	0.548
	ENMLR			24.85	0.773		0.595	24.72	0.319	0.565
	ESR			20.26	0.630		0.516	21.77	0.376	0.613
	XGB	ES	Trainng Set	7.19	6.057		4.553	5.33	0.581	0.762
	RF			6.00	5.051		3.150	3.69	0.707	0.841
	SVR			6.26	5.275		3.255	3.71	0.697	0.835
	ENMLR			7.95	6.692		5.298	6.15	0.490	0.700
	ESR			6.10	5.136		3.299	3.90	0.699	0.836
	XGB		Testing Set	8.27	7.045		5.437	6.34	0.484	0.696
	RF			6.62	5.640		3.442	3.82	0.674	0.821
	SVR			6.94	5.917		3.623	4.04	0.667	0.817
	ENMLR			8.20	6.982		5.705	6.57	0.501	0.708
	ESR			6.45	5.494		3.328	3.82	0.687	0.829
	XGB	SL	Trainng Set	21.19	0.587		0.413	13.87	0.789	0.888
‘Atabaki’	RF	SL	Trainng Set	8.46	0.234		0.153	5.25	0.966	0.983
	SVR			8.57	0.237		0.148	5.08	0.964	0.982
	ENMLR			24.00	0.665		0.470	16.18	0.729	0.854
	ESR			11.05	0.306		0.214	7.66	0.953	0.976
	XGB		Testing Set	20.91	0.596		0.469	16.22	0.759	0.871
	RF			11.07	0.316		0.205	6.35	0.935	0.967
	SVR			11.36	0.324		0.205	6.38	0.933	0.966
	ENMLR			21.16	0.617		0.498	17.88	0.729	0.854
	ESR			10.60	0.302		0.199	6.78	0.935	0.967

*SVR* Support Vector Regression, *RF* Random Forest, *XGB* Extreme Gradient Boosting, *ENMLR* Elastic Net Multivariate Linear Regression, *ESR* Ensemble Stacking Regression, *R* coefficient of determination, *RRMSE* Relative Root Mean Square Error, *RMSE* Root Mean Square Error, *MAPE* Mean Absolute Percentage Error, *PR* proliferation rate, *SL* shoot length, *LN* leave number, and *ES* explant survival

**Table 5:** Statistical evaluation of the constructed models for the micropropagation of the pomegranate cultivar ‘Faroogh’

Cultivar	Model	Study parameter	Data sets	Performance criteria					
				RRMSE (%)	RMSE	MAE	MAPE (%)	R^2^	R
‘Faroogh’	XGB	LN	Trainng Set	5.33	1.079	0.801	3.89	0.545	0.738
	RF			4.78	0.969	0.749	3.65	0.645	0.803
	SVR			4.52	0.915	0.692	3.39	0.677	0.823
	ENMLR			5.78	1.170	0.918	4.46	0.462	0.680
	ESR			4.49	0.909	0.699	3.43	0.676	0.822
	XGB		Testing Set	6.01	1.225	0.926	4.51	0.498	0.706
	RF			5.11	1.041	0.834	4.05	0.671	0.819
	SVR			4.85	0.989	0.801	3.92	0.684	0.827
	ENMLR			6.60	1.345	1.060	5.17	0.393	0.627
	ESR			4.60	0.938	0.730	3.58	0.711	0.843
	XGB	PR	Trainng Set	12.86	0.562	0.394	11.82	0.212	0.460
	RF			12.82	0.461	0.399	11.95	0.220	0.469
	SVR			13.42	0.483	0.449	13.41	0.172	0.415
	ENMLR			13.54	0.487	0.432	12.97	0.127	0.356
	ESR			12.86	0.462	0.394	11.87	0.213	0.462
	XGB		Testing Set	14.29	0.518	0.427	13.30	0.182	0.427
	RF			14.17	0.514	0.430	13.37	0.199	0.446
	SVR			14.54	0.527	0.470	14.63	0.208	0.456
	ENMLR			14.38	0.521	0.444	13.95	0.186	0.431
	ESR			14.14	0.512	0.418	13.10	0.199	0.446
	XGB	ES	Trainng Set	6.26	5.456	4.646	5.42	0.514	0.717
	RF			5.42	4.720	4.054	4.76	0.637	0.798
	SVR			5.64	4.912	4.366	5.01	0.634	0.796
	ENMLR			6.87	5.981	5.188	6.00	0.417	0.646
	ESR			5.47	4.769	4.094	4.82	0.630	0.794
	XGB		Testing Set	6.32	5.516	4.582	5.40	0.514	0.717
	RF			4.92	4.291	3.778	4.46	0.699	0.836
	SVR			5.07	4.424	4.025	4.61	0.711	0.843
	ENMLR			6.67	5.822	4.920	5.71	0.448	0.669
	ESR			4.84	4.228	3.711	4.39	0.709	0.842
	XGB	SL	Trainng Set	11.72	0.375	0.279	8.49	0.699	0.836
‘Faroogh’	RF			8.46	0.271	0.184	5.40	0.843	0.918
	SVR			8.95	0.286	0.199	6.02	0.828	0.910
	ENMLR			12.54	0.401	0.295	8.99	0.654	0.809
	ESR			8.84	0.283	0.190	5.66	0.830	0.911
	XGB		Testing Set	13.77	0.449	0.339	10.16	0.707	0.841
	RF			9.94	0.259	0.177	4.88	0.937	0.968
	SVR			7.33	0.239	0.175	5.11	0.927	0.963
	ENMLR			15.40	0.503	0.380	11.39	0.624	0.790
	ESR			7.27	0.237	0.164	4.66	0.941	0.970

*SVR* Support Vector Regression, *RF* Random Forest, *XGB* Extreme Gradient Boosting, *ENMLR* Elastic Net Multivariate Linear Regression, *ESR* Ensemble Stacking Regression, *R* coefficient of determination, *RRMSE* Relative Root Mean Square Error, *RMSE* Root Mean Square Error, *MAPE* Mean Absolute Percentage Error, *PR* proliferation rate, *SL* shoot length, *LN* leave number, and *ES* explant survival

**Table 6:** Statistical evaluation of the constructed models for the micropropagation of the pomegranate cultivar ‘Shirineshahvar’

Cultivar	Model	Study parameter	Data Sets	Performance Criteria					
				RRMSE (%)	RMSE	MAE	MAPE (%)	R^2^	R
‘Shirineshahvar’	XGB	LN	Trainng Set	6.63	1.156	0.932	5.64	0.616	0.785
	RF			4.86	0.849	0.702	4.11	0.792	0.890
	SVR			5.04	0.879	0.738	4.33	0.783	0.885
	ENMLR			7.43	1.297	0.957	5.96	0.516	0.718
	ESR			4.91	0.857	0.706	4.14	0.789	0.888
	XGB		Testing Set	6.66	1.157	0.896	5.67	0.740	0.860
	RF			4.68	0.812	0.650	3.84	0.863	0.929
	SVR			4.67	0.811	0.653	3.92	0.863	0.929
	ENMLR			8.40	1.457	1.081	7.03	0.593	0.770
	ESR			4.64	0.805	0.644	3.81	0.865	0.930
	XGB	PR	Trainng Set	19.19	0.496	0.358	17.20	0.650	0.806
	RF			20.49	0.530	0.365	19.65	0.627	0.792
	SVR			20.00	0.517	0.355	18.39	0.654	0.809
	ENMLR			22.99	0.594	0.463	23.54	0.503	0.709
	ESR			19.04	0.492	0.375	19.26	0.656	0.810
	XGB		Testing Set	21.86	0.546	0.407	20.98	0.630	0.794
	RF			23.92	0.598	0.431	25.86	0.590	0.768
	SVR			22.35	0.559	0.398	22.64	0.638	0.799
	ENMLR			24.00	0.600	0.503	28.14	0.584	0.764
	ESR			21.07	0.526	0.406	22.45	0.677	0.823
	XGB	ES	Trainng Set	11.08	5.825	4.768	9.20	0.354	0.595
	RF			10.58	5.563	4.525	8.71	0.413	0.643
	SVR			10.86	5.706	4.866	9.48	0.387	0.622
	ENMLR			12.35	6.490	5.179	9.98	0.307	0.554
	XGB		Testing Set	11.08	5.769	4.821	9.46	0.388	0.623
	RF			11.26	5.862	4.834	9.52	0.361	0.601
	SVR			11.73	6.107	5.233	10.37	0.338	0.581
	ENMLR			12.49	6.501	5.222	10.26	0.375	0.612
	ESR			11.05	5.754	4.752	9.44	0.391	0.625
	XGB	SL	Trainng Set	5.14	0.084	0.063	4.07	0.845	0.919
‘Shirineshahvar’	RF	SL	Trainng Set	4.58	0.075	0.058	3.76	0.870	0.933
	SVR			4.67	0.076	0.059	3.84	0.867	0.931
	ENMLR			6.03	0.098	0.073	4.72	0.774	0.880
	ESR			4.75	0.077	0.058	3.78	0.861	0.928
	XGB		Testing Set	4.97	0.081	0.063	3.96	0.845	0.919
	RF			4.54	0.074	0.057	3.64	0.867	0.931
	SVR			4.64	0.075	0.055	3.52	0.857	0.926
	ENMLR			6.60	0.108	0.081	5.11	0.717	0.847
	ESR			4.45	0.072	0.055	3.48	0.870	0.933

*SVR* Support Vector Regression, *RF* Random Forest, *XGB* Extreme Gradient Boosting, *ENMLR* Elastic Net Multivariate Linear Regression, *ESR* Ensemble Stacking Regression, *R* coefficient of determination, *RRMSE* Relative Root Mean Square Error, *RMSE* Root Mean Square Error, *MAPE* Mean Absolute Percentage Error, *PR* proliferation rate, *SL* shoot length, *LN* leave number, and *ES* explant survival

**Table 7:** Ranking of the best-performing ML models for growth parameters of pomegranate

Model	Study parameter	‘Atabaki’		‘Faroogh’		‘Shirineshahvar’	
		GPI	Rank	GPI	Rank	GPI	Rank
XGB	LN	− 1.674	4	− 2.562	4	− 3.040	4
RF		0.647	2	0.000	3	0.032	2
SVR		0.000	3	0.525	2	0.004	3
ENMLR		− 4.171	5	− 4.688	5	− 5.911	5
ESR		**1.829**	**1**	**1.312**	**1**	**0.089**	**1**
XGB	PR	0.980	2	− 1.199	3	0.574	2
RF		0.738	3	0.567	2	− 2.616	4
SVR		− 2.326	4	− 2.121	4	0.255	3
ENMLR		− 3.801	5	− 2.104	5	− 3.807	5
ESR		**1.383**	**1**	**1.182**	**1**	**1.911**	**1**
XGB	ES	− 4.870	5	− 3.813	4	0.818	2
RF		0.573	2	0.483	2	− 1.501	3
SVR		0.000	3	0.085	3	− 2.966	5
ENMLR		− 4.814	4	− 5.240	5	− 2.507	4
ESR		**0.933**	**1**	**0.748**	**1**	**0.973**	**1**
XGB	SL	− 5.158	4	− 4.058	4	− 0.913	4
RF		0.108	2	0.092	3	0.193	2
SVR		0.035	3	0.405	2	0.150	3
ENMLR		− 5.782	5	− 5.380	5	− 5.487	5
ESR		**0.180**	**1**	**0.619**	**1**	**0.513**	**1**

SVR: Support Vector Regression, RF: Random Forest, XGB: Extreme Gradient Boosting, ENMLR: Elastic Net Multivariate Linear Regression, ESR: Ensemble Stacking Regression, GPI: Global Performance Indicator, PR: proliferation rate, SL: shoot length, LN: leave number, and ES: explant survival

Bold values have mentioned the biggest and best values, respectively.

### Optimization process via non-dominated sorting genetic algorithm-II

The NSGA-II algorithm, as multi-objective evolutionary optimization, was linked to the ESR model which was identified as the most accurate algorithm. ESR-NSGA-II algorithm has successfully determined the optimal values for four growth parameters (LN, PR, ES, and SL) in response to different concentrations of PGRs. The results of the ESR-NSGA-II algorithm are summarized in Table 8. In the ‘Atabaki’ cultivar, the ESR-NSGA-II algorithm identified that the culture medium supplemented with 0.750 mg/L ZT along with, 0.50 mg/L GA_3_, resulted in the most significant improvements in growth parameters. Specifically, this combination treatment displayed the best outputs with 18.18 LN, 3.47 PR, 84.21% ES, and 2.74 cm SL. For the ‘Faroogh’ cultivar, the optimization algorithm determined that the culture medium supplemented with 0.654 mg/L ZT along with, 0.329 mg/L GA_3_ were the optimal input variables to achieve the best outputs with 19.76 LN, 3.84 PR, 85.49% ES, and 3.32 cm SL. In the ‘Shirineshahvar’ cultivar, the culture medium supplemented with 0.705 mg/L ZT, combined with 0.347 mg/L GA_3_, were the significant input variables to achieve the best outputs with 18.77 LN, 3.22 PR, 56.39% ES, and 1.86 cm SL (Table 8).

**Table 8:** Optimization of pomegranate cultivars and different concentrations of ZT, and GA_3_ according to the ESR-NSGA-II algorithm to obtain the best plant growth parameters

Input variables			Predicted LN	Predicted PR	Predicted ES (%)	Predicted SL (cm)
Cultivar	GA_3_	ZT				
‘Atabaki’	0.500	0.750	18.18	3.47	84.21	2.74
‘Faroogh’	0.329	0.654	19.76	3.84	85.49	3.32
‘Shirineshahvar’	0.347	0.705	18.77	3.22	56.39	1.86

*GA*_*3*_ gibberellic acid, *ZT* zeatin, *PR* proliferation rate, *SL* shoot length, *LN* leave number, and *ES* explant survival

## Discussion

The success of in vitro plant tissue culture strongly depends on several external and internal factors, including environmental conditions, PGRs types, culture medium composition, and gelling agents, and genotype [18]. The application of PGRs, particularly cytokinin and auxin, are commonly used to optimize protocols for in vitro tissue culture and shoot regeneration [17, 54, 55]. Auxin increases the susceptibility of apical meristem cells that are less mitotically active cells to cytokinin [56], while cytokinin promotes cell proliferation, including cell division and shoot elongation [10]. In the case of pomegranate, which is a recalcitrant woody plant for in vitro culture, the optimization of type and concentration of PGRs, as well as their interactions, play a crucial role [8, 57–59].

In previous studies to efficiently multiply various pomegranate species, it has been reported that integrating BAP with or without NAA at specific concentrations ranging from 0.4 to 2 mg/L for BAP and 0.5 to 1 mg/L for NAA, has proven effective [57]. However, it is important to note that the results of these studies are often specific to particular cultivars and cannot be universally applied. The optimization of PGR concentrations is necessary due to genetic factors and complexities associated with the oxidation of phenols in explants and culture media, which can lead to tissue death. Furthermore, pomegranate tissue culture protocols are highly dependent on the cultivar and may differ due to variations in uptake rates, translocation rates, or metabolic processes within the meristematic regions of the plant. Additionally, cytokinin metabolism plays a crucial role, as cytokinins may undergo degradation or conjugation with sugars or amino acids, leading to the formation of biologically inert compounds, as reported by Desai et al. [60].

Although ZT has been recognized as highly effective in promoting shoot proliferation in various plant species [61–63], its use in pomegranate tissue culture has limited compared to other cytokinins. Similarly, the use of GA_3_ in shoot proliferation, particularly in recalcitrant woody trees like pomegranate, has received limited attention. However, several studies have demonstrated that the interaction between cytokinins with GA_3_ can improve the development of shoot/root apical meristems [8, 64, 65]. This study introduces a new shoot proliferation protocol for pomegranate cultivars, which utilizes a combination of ZT and GA_3_. The results demonstrate the remarkable efficacy of this combination in stimulating shoot development compared to using BAP alone. Notably, the treatment involving the highest concentration of both ZT and GA_3_ exhibited the most significant growth response, highlighting its effectiveness. Additionally, GA_3_ enhanced shoot regeneration and increased the ES% of all three tested pomegranate cultivars when combined with cytokinins and auxins. Although limited reports exist on the effect of ZT on shoot proliferation in pomegranate, Naik et al. [66] reported significant improvements in regeneration frequency and shoot growth by adding zeatin riboside (ZR) to the culture medium. The analysis of current study also highlighted that different pomegranate cultivars exhibited different reactions to the same culture medium, despite their close genetic relationship. It is noteworthy that the ‘Faroogh’ cultivar exhibited the highest growth responses among the three cultivars investigated. However, the ‘Shirineshahvar’ cultivar displayed higher recalcitrant to shoot proliferation compared to the other cultivars. This could be attributed to variations in the concentration of endogenous phytohormones within the plants and their interaction with the applied exogenous PGRs in the culture of explants [67].

Developing and optimizing tissue culture protocols is a complex task that poses significant challenges to the field as a whole. The multifactorial nature of in vitro culture processes makes them difficult to understand and interpret using traditional statistical approaches such as ANOVA, t-tests, correlation, and regression, specifically when the variables investigated are nonlinear, noisy, complex, and vague in nature [68]. The knowledge derived from MLs, as complex mathematical tools, offer promise in understanding and interpreting the intricate, nonlinear relationships within datasets. ML models have demonstrated superior predictive power over traditional statistical methodologies when analyzing unpredictable variables and big dataset. Despite the advantages of ML, uncertainty in ML outcomes remains a major constraint in its application [69]. Uncertainty in ML studies arises from three primary sources: data quality, the sample of data collected from the domain, and model fitting [70]. To avoid uncertainties, researchers have recommended the application of different ML algorithms [69, 70]. In this study, five ML approaches (XGB, RF, SVR, ESR, and ENMLR) were employed for modeling the effects of various parameters (PGRs) on in vitro shoot proliferation of pomegranate. While similar performance was observed across the ML models in predicting pomegranate shoot multiplication, the results of the GPI analysis indicated that the ESR model stood out as the best performer. It exhibited robustness and superior predictive accuracy in both the training and testing subsets. It is worth noting that there is a lack of specific investigations regarding the use of the ESR algorithm in the field of plant tissue culture. Nonetheless, numerous studies in other scientific disciplines have demonstrated the robust performance of the ESR model in various prediction tasks [71, 72]. In recent research has shown that integrating optimization algorithms, particularly NSGA-II, with ML models can provide valuable insights and effective utilization of the models. The application of NSGA-II in conjunction with ML enables the answering of "How to get" questions by identifying the optimal culture medium that simultaneously improves multiple desired parameters for the studied parameters [18, 73]. In the current research, the ESR was linked to the NSGA-II algorithm as a computational forecasting approach for predicting and identifying critical factors affecting the in vitro proliferation stage of pomegranate cultivars. The successful application of optimization algorithms, especially NSGA-II, in the field of plant tissue culture has already been accomplished [31]. Additionally, various ML algorithms based on different optimization algorithms have shown promising results in modeling and predicting optimal plant tissue culture media for other fruit tree species such as kiwi berry [18], pear [74], prunus [15], pistachio rootstocks [74], and Persian walnut [10]. The outcomes obtained through the ESR-NSGA-II method accurately predicted that the highest plant growth responses would be achieved by supplementing the culture medium with 0.750 mg/L ZT, and 0.500 mg/L GA_3_ for the ‘Atabaki’ cultivar, 0.654 mg/L ZT, and 0.329 mg/L GA_3_ for the ‘Faroogh’ cultivar, and 0.705 mg/L ZT, and 0.347 mg/L GA_3_ for the ‘Shirineshahvar’ cultivar. Overall, the ESR-NSGA-II algorithm revealed that the interaction between genotype and different concentrations of PGRs caused the most significant influence on pomegranate shoot proliferation. These findings are consistent with a study by Sadat-Hoseini et al. [10], which employed ML approaches to model growth parameters of in vitro Persian walnut using different concentrations of BAP, tidiazuran (TDZ), and indole butyric acid (IBA), and reported that the genotype-PGR interaction plays a crucial role in the proliferation of Persian walnut.

To the best of the author’s knowledge, this study represents the first investigation examining the specific effects of ZT and GA_3_, as well as their interactions, in enhancing the efficiency of pomegranate tissue culture protocol, especially with the studied pomegranate cultivars on in vitro conditions for enhancing growth parameters. While previous studies have reported in vitro shoot proliferation success of different pomegranate cultivars, the focus on the specific combination of ZT and GA_3_, and their interactions effects, is a novel aspect of this research. By evaluating the influence of these growth regulators on growth parameters, this study contributes to the advancement of pomegranate tissue culture techniques.

## Conclusion

In vitro shoot proliferation is a multifactorial and complex process influenced by various interacting factors. So, to evaluate the extensive datasets and optimize the pomegranate protocol, ML techniques such as RF, SVR, XGB, ESR, and ENMLR were employed as promising alternatives to traditional statistical methods. Based on our results, ESR-NSGA-II exhibited superior accuracy and efficacy in studying pomegranate growth responses to multivariable stimuli in vitro and optimizing the pomegranate protocol. Furthermore, the in vitro responses of pomegranate were found to be positively influenced by the concentrations of PGRs (ZT and GA_3_) and their interaction. Moreover, the optimization of in vitro condition of pomegranate was strongly depended on the specific cultivar. Specifically, the ‘Shirineshahvar’ cultivar demonstrated as a recalcitrant cultivar to in vitro shoot proliferation compared to other cultivars, while the ‘Faroogh’ cultivar exhibited the highest growth and shoot development. The main objective of the current research was to provide a reliable and robust technology, ESR-NSGA-II based on soft computing methodology, to provide new insight into the crucial factors that impact the growth parameters of pomegranate cultivars cultured in vitro.

## Data Availability

The authors confirm that the datasets analyzed during the current study are available from the corresponding author on reasonable request.
